# Impact of a digital application on HbA1c levels in people with diabetes: a randomized controlled trial

**DOI:** 10.3389/fdgth.2025.1544668

**Published:** 2025-06-30

**Authors:** Lena Roth, Nico Steckhan, Peter E. H. Schwarz

**Affiliations:** Department for Prevention and Care of Diabetes, Department of Medicine III, Faculty of Medicine Carl Gustav Carus, Technische Universität Dresden, Dresden, Germany

**Keywords:** glycemic control, digital health application (DiHA/DiGA), insulin-treated diabetes mellitus, diabetes care technology, diabetes self-management, blood glucose tracking, HbA1c reduction, digital diabetes management

## Abstract

**Aims:**

Digital applications have the potential to enhance diabetes management, particularly in patients treated with insulin. This study aims to evaluate the impact of a digital application on self-management (ESYSTA, Germany), expressed in a change in HbA1c levels, in people with diabetes treated with insulin.

**Materials and methods:**

A randomized controlled, multicentric trial was conducted in 204 people with diabetes (60% type 2 diabetes) treated with insulin to assess the efficacy of ESYSTA. Participants were randomly assigned to either the intervention group (IG) using ESYSTA in addition to the German standard of Care (SoC) according to the German disease management programs (DMP) for 6 months or a control group (CG) receiving SoC only. The primary endpoint was the change in HbA1c levels. Secondary endpoints included well-being and diabetes-related distress.

**Results:**

A clinically relevant reduction in HbA1c levels of on average −0.48% points (−0.66; −0.29) was observed in the IG after 6 months. Compared to the CG, this reduction was more pronounced, especially in the per-protocol sample [mean difference, −0.28% points; 95% CI (−0.54; −0.02)]. Improvements in the IG in further secondary endpoints, such as well-being or diabetes-related distress, indicate enhanced overall glycemic control and patient satisfaction in the IG.

**Conclusions:**

The use of ESYSTA improved HbA1c levels and other secondary outcomes in people with diabetes who are treated with insulin in comparison with German SoC in the context of DMP. These findings support the integration of digital tools in routine diabetes care to optimize patient outcomes.

**Clinical Trial Registration:**

https://drks.de/search/de/trial/DRKS00025996, identifier DRKS00025996.

## Introduction

Diabetes mellitus is a chronic, non-communicable disease. Due to defects in insulin secretion, insulin action, or both, it causes chronic hyperglycemia, i.e., raised levels of blood glucose (1, 2). It is associated with several micro- and macrovascular risks and comorbidities and is one of the leading causes of death and disability-adjusted life years worldwide (2–4). The prevalence is continuously increasing, currently affecting approximately 10% of the population globally (2). In Germany, the prevalence is reported as 9%−10% (2, 5). Diabetes prevalence substantially increases with age, reaching levels of over 20% in the elderly population (5).

The most common type of diabetes mellitus, which accounts for approximately 90% of diabetes cases worldwide, is type 2 (T2D). In contrast to type 1 diabetes mellitus (T1D), T2D develops slowly and manifests later in life, as it is strongly linked to lifestyle factors such as high-calorie diets, sedentary behavior, and smoking (2, 6). Overweight, obesity, high blood pressure, and hyperlipidemia are further increasing the risk of T2D (2, 6). In consequence, the initial treatment focuses on lifestyle modifications with the gradual addition of pharmacotherapy escalated by a stepwise initiation of insulin treatment (7, 8). Approximately 20%–36% of T2D patients in Germany are treated with insulin (5, 9). T1D, on the other hand, is an autoimmune reaction that requires immediate and lifelong insulin substitution as soon as it manifests, mostly already during childhood or early adulthood (7).

The chronic nature of diabetes requires affected patients to continuously manage their metabolic condition, including their medication and lifestyle, independent of medical care. Especially, digital technologies are seen as a promising therapeutic intervention to improve blood glucose control, thus reducing the burden of disease through optimized self-management of the patient (10, 11). With the Digital Healthcare Act (2020) Evidence–based treatments with digital health applications (DiGA) in the form of medical devices can be fully reimbursed by health insurance companies in Germany as the first country worldwide (12). To be eligible for prescription and reimbursement, DiGA need to fulfill quality, data security, and risk requirements and need to demonstrate a positive healthcare effect in a comparative study. Those criteria are reviewed, checked, and approved by the German Federal Institute for Drugs and Medical Devices (BfArM) within the “DiGA Fast-Track” procedure (12).

This study aims to assess the effectiveness of the digital application ESYSTA (Emperra GmbH E-Health Technologies, Germany) to ultimately provide the necessary evidence for certification as an official DiGA in Germany, following the “DiGA Fast-Track evaluation” as a guideline for the approval.

## Materials and methods

### Study design and participants

The DAVOS trial (EValuation Of the Impact of ESYSTA on HbA1c in Patients with Type 1 and Type 2 Diabetes mellitus in DAily practice) was an open-label, 6-month prospective, multicentric (21 study centers), randomized, controlled study with two arms: an intervention group (IG) that used ESYSTA for a period of 6 months in addition to SoC in Germany and a control group (CG) that was treated according to SoC in Germany without any additional intervention. The study received approval from the relevant ethics committee (10.02.2022, BO-EK-534112021); after assessment of eligibility (Table 1), participants provided informed consent prior to enrollment.

**Table 1:** Inclusion and exclusion criteria.

Inclusion criteria	Exclusion criteria
• Diabetes mellitus type 1 or 2 • ≥18 years old • Treated with insulin • HbA1c at baseline ≥7.5% and ≤11.0% • Not having used the investigated DiGA in the past 12 months • Willing to use a smart glucometer and/or smart insulin pen • Internet access, smartphone/computer compatible with the DiGA • Digital literacy to use a smartphone adequately	• Using other apps linked to a blood glucose meter or using an insulin pump or continuous glucose monitoring • Having used the investigated DiGA in the past 12 months • <18 years old • Impairments (also mental impairments, e.g., psychotic disorders) • Patients with home nursing (assisting with BG testing) • Current participation in a weight loss program • Current participation in another study • Steroid therapy within the past three months (no exclusion criterion if used topically or inhaled less than five times a week) • Blood pressure ≥200 mm Hg at screening • BMI ≥40 kg/m^2^ • Anemia (according to the WHO definition) • Glomerular filtration rate ≤40 ml/min • Current or planned pregnancy, breastfeeding women • Alcohol or drug abuse (within the past three months) • Employees of the Emperra GmbH E-Health Technologies (or other institutions being involved in the trial)

BMI, body mass index; WHO, World Health Organization.

### Intervention

The SoC for diabetes in Germany is regulated and overseen by the federal state in structured treatment plans based on evidence-based medicine, i.e., the Disease Management Programmes (DMP) for T1D or T2D. It includes regular monitoring of blood glucose levels, HbA1c levels, blood pressure, cholesterol, and kidney function parameters to manage and prevent complications of diabetes. A particular focus is on patient education, lifestyle modifications, and regular follow-ups with healthcare providers to ensure adherence to treatment plans.

ESYSTA is a digital health solution developed by Emperra GmbH E-Health Technologies to support people with diabetes in improving their disease management. Relevant health data, such as glucose readings and insulin dosages, can be transferred to the web-based ESYSTA portal and the ESYSTA app automatically by using devices connected via interoperability supporting interfaces, including smart pens or blood glucose meters, or can be entered manually. Special algorithms continuously analyze and evaluate the data, e.g., a profile comparison of limit values, any insulin used, daily blood glucose curves (3- or 7-day view), and insulin doses. A traffic light display provides a quick overview of critical values or incorrect doses. The analyzed and visualized health data can be Accessed via the ESYSTA portal and the ESYSTA app to help patients make informed decisions about their diabetes management. To improve communication and interaction with the healthcare provider, the patient may also grant the medical care team access to the ESYSTA portal, which ultimately would open up the possibility of remote treatment and of monitoring the quality-assured usage on patient's side. Finally, the software supports patients with automatic empowerment messages and suggests individually tailored prevention offers. A detailed description of the functions of the ESYSTA app and portal, as well as screenshots, can be found in Supplementary Table S1 and Figures S1–S3.

### Study procedure

The study protocol, including detailed information about recruitment, randomization, and study procedures, was described previously (13). To adapt to the changing requirements within the Fast-Track procedure, minor deviations had to be made including the final statistical analysis plan that was adapted before data were viewed and analyzed. The study was registered in the German Register of Clinical Trials (DRKS) with the number DRKS00025996.

### Outcomes

The primary endpoint was defined as the change from baseline of HbA1c levels (in %) after 6 months. HbA1c levels were measured by health care professionals according to the SoC in Germany.

Secondary objectives aimed to evaluate the effect of ESYSTA in regard to diabetes-related distress [Problem Areas in Diabetes Questionnaire (PAID)] and well-being (World Health Organization-Five questionnaire; WHO-5). PAID assesses diabetes-related emotional distress with 20 items on a scale from 0 (no distress) to 100 (serious distress) (14). Scores above 40 indicate “emotional burnout” (15). The WHO-5 measures well-being on a scale from 0 to 25, with higher scores indicating better well-being (16). Scores below 13 indicate impaired well-being (17).

To evaluate additional beneficial effects of ESYSTA, e.g., the proportion of patients achieving HbA1c treatment goals [defined as HbA1c levels below 6.5% and 7.0% (3, 8)], patient self-management (SDSCA-G questionnaire, using the overall scale for assessment) and quality of life (EQ-5D-5L questionnaire) were defined as exploratory endpoints. The SDSCA-G consists of 11 items in the area of diabetes-related self-management, i.e., nutrition, physical activity, blood glucose control, and foot care, answered on a scale of 0–7 (equal to the number days in the last week in which a certain activity was performed) (18, 19). EQ-5D-5L measures quality of life and is divided into five dimensions, with levels from 1 (no problem) to 5 (extreme problems). To estimate the change in quality of life/health status, the index of quality of life was considered. For this, a health state profile for each patient was provided according to the EQ-5D-5L user guide (20). This profile was then substituted by an index from the German validated value set, with 1 indicating the best health states and no problems in any domain (21).

### Statistical analysis

For the data analysis, the software R version 4.3. was used (22). The primary confirmatory analysis was an intention-to-treat (ITT) analysis, including all randomized study subjects independent of protocol deviations.

For the ITT analysis, missing endpoint values were imputed with multiple imputation using the R function *RefBasedMI* and the implemented copy increments to reference (CIR) methods, with the CG defined as the reference group. All covariates that were part of the regression model were also used as covariates for imputation. The differences in endpoints at 3 and 6 months were calculated after imputing the raw values at each time point.

A second per-protocol analysis (PP) was performed as a sensitivity analysis, including all randomized subjects who completed the whole study period (complete cases) and who adhered to the protocol.

Linear (mixed) models were fitted for each endpoint to each of the resulting imputed data sets and pooled using multivariate Wald tests in the R package mitml (7, 8). For the primary endpoint, the dependent variable was the change in HbA1c levels compared with baseline (in % points). The independent variables were the within-group factor *time* (3 and 6 months) and the between-group factor *group* (IG and CG), included in the model as interacting factors. To control for potential confounding, gender, indication, and baseline HbA1c levels were included as covariates. Moreover, a random intercept for each participant was included.

Because the secondary endpoints were assessed only after 6 months, linear models were fitted and did not include a within-group factor. For the secondary endpoints, the dependent variable was the change in well-being or diabetes-related distress at 6 months compared with baseline. As in the model for the primary endpoint, potential confounders were included as covariates, including the baseline value for the respective endpoint.

Hypotheses were tested using *a priori* defined contrasts (H1, H3, H4) and a one-sample *t*-test against less than or equal to −0.4 (H2). Due to multiple testing, Benjamin–Hochberg FDR correction for the hypotheses regarding the primary endpoint was performed. For the secondary endpoints, hierarchical testing was applied following the sequence shown in Table 2.

**Table 2:** Hypotheses related to the primary and secondary endpoints.

Endpoint	Hypotheses
Primary endpoint: changes in HbA1c (in %)	H1: difference in change from baseline after 6 months of treatment in HbA1c between ESYSTA and standard diabetes care. H2: the use of ESYSTA over 6 months has a clinically meaningful impact on lowering the HbA1c by at least 0.4% points.
First secondary endpoint: changes in diabetes-associated distress (PAID)	H3: difference in change from baseline after 6 months of treatment in diabetes-associated distress assessed by the PAID between ESYSTA and standard
Second secondary endpoint: changes in well-being (WHO-5)	H4: difference in change from baseline after 6 months of treatment in well-being assessed by the WHO-5 between ESYSTA and standard diabetes care.

Hypotheses yielding *p* < 0.05 are considered significant. As an additional effect size measurement, Cohen’s *d* was calculated based on the *t* values from the hypothesis tests.

Additional exploratory endpoints were only analyzed based on the ITT sample, using linear (mixed) models analogous to the primary or secondary endpoints, depending on the frequency of assessment.

## Results

### Participant characteristics

**Figure 1:**
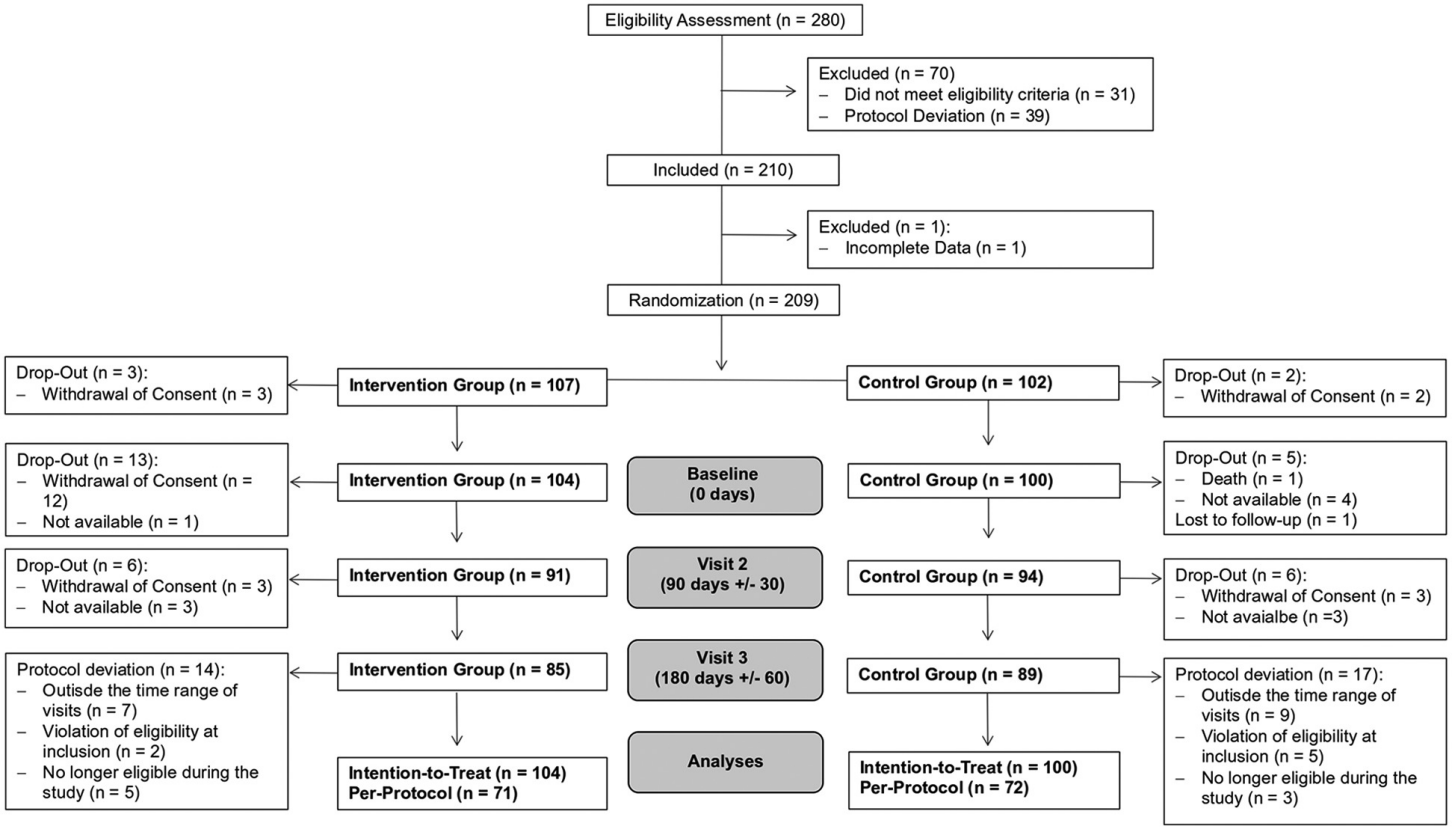
Patient flow.

In total, 280 people with diabetes were screened for eligibility, of whom 210 fulfilled the inclusion criteria. However, only 209 provided the data needed for randomization. After randomization, three patients of the IG and two of the CG immediately dropped out and are not included in the analyses. Overall, the ITT sample consists of 104 participants in the IG and 100 in the CG. In total, 18.3% of the IG and 11% of the CG participants dropped out. Due to protocol deviations, additional participants, apart from the dropouts, had to be excluded, leaving 143 participants (71 in the IG and 72 in the CG) who are included in the PP analyses. The detailed patient flow is shown in Figure 1.

Of the final 204 participants, 61.8% were male and 59.8% had T2D while 40% had T1D. The average age was 55.2 ± 13.7 years (M ± SD). The average baseline HbA1c value was 8.47% ± 0.86. Table 3 gives an overview of the baseline characteristics and measures for the primary, secondary, and exploratory endpoints, showing that the groups were comparable at baseline. Detailed information on baseline values of the self-management (SDSCA-G) and quality-of-life (EQ-5D-5L) subscales, as well as baseline characteristics of the PP population, is shown in the Supplementary Tables S3–S5. The ITT and PP populations are also comparable at baseline, with the PP population consisting of a slightly higher proportion of people with T2D.

**Table 3:** Demographic and endpoint characteristics of the randomized ITT population at baseline.

	IG	CG	Overall
	(*n* = 104)	(*n* = 100)	(*N* = 204)
Age			
Mean (SD)	54.0 (14.2)	56.4 (13.2)	55.2 (13.7)
Median [min, max]	57.0 [18.0, 84.0]	59.0 [19.0, 91.0]	58.0 [18.0, 91.0]
Gender			
Male	63 (60.6%)	63 (63.0%)	126 (61.8%)
Female	41 (39.4%)	37 (37.0%)	78 (38.2%)
Type of diabetes			
T1D	40 (38.5%)	42 (42.0%)	82 (40.2%)
T2D	64 (61.5%)	58 (58.0%)	122 (59.8%)
Years since diabetes diagnosis			
Mean (SD)	14.2 (10.3)	15.9 (9.99)	15.0 (10.2)
Median [min, max]	13.0 [0, 51.0]	15.5 [0, 48.0]	13.5 [0, 51.0]
HbA1c (in %)			
Mean (SD)	8.47 (0.86)	8.46 (0.81)	8.47 (0.83)
Median [min, max]	8.20 [7.50, 11.0]	8.30 [7.40, 10.8]	8.25 [7.40, 11.0]
Well-being			
Mean (SD)	14.9 (5.47)	15.5 (5.74)	15.2 (5.60)
Median [min, max]	16.0 [2.00, 24.0]	16.0 [2.00, 25.0]	16.0 [2.00, 25.0]
Diabetes-related distress			
Mean (SD)	16.8 (16.9)	13.8 (14.0)	15.3 (15.6)
Median [min, max]	13.1 [0, 81.3]	8.75 [0, 70.0]	10.0 [0, 81.3]
Weight (in kg)			
Mean (SD)	89.8 (19.5)	93.2 (20.4)	91.5 (20.0)
Median [min, max]	89.0 [52.0, 150]	91.5 [57.5, 162]	89.9 [52.0, 162]
BMI (in kg/m^2^)			
Mean (SD)	29.4 (5.39)	30.9 (6.15)	30.1 (5.81)
Median [min, max]	28.9 [19.6, 47.8]	30.7 [18.3, 52.9]	30.0 [18.3, 52.9]
Waist circumference (in cm)			
Mean (SD)	107 (15.0)	109 (18.4)	108 (16.7)
Median [min, max]	107 [74.0, 145]	106 [72.0, 152]	107 [72.0, 152]
Missing	7 (6.7%)	10 (10.0%)	17 (8.3%)
SDSCA-G (overall)			
Mean (SD)	4.02 (0.995)	4.15 (1.05)	4.08 (1.02)
Median [min, max]	4.05 [1.60, 6.20]	4.10 [1.30, 6.80]	4.10 [1.30, 6.80]

BMI, body mass index; CG, control group; HbA1c, glycated hemoglobin; IG, intervention group; Min, minimum; Max, maximum; SD, standard deviation; T1D/T2D, diabetes mellitus type 1/2; SDSCA-G, German version of the summary of diabetes self-care activities.

### Changes in HbA1c levels

**Figure 2:**
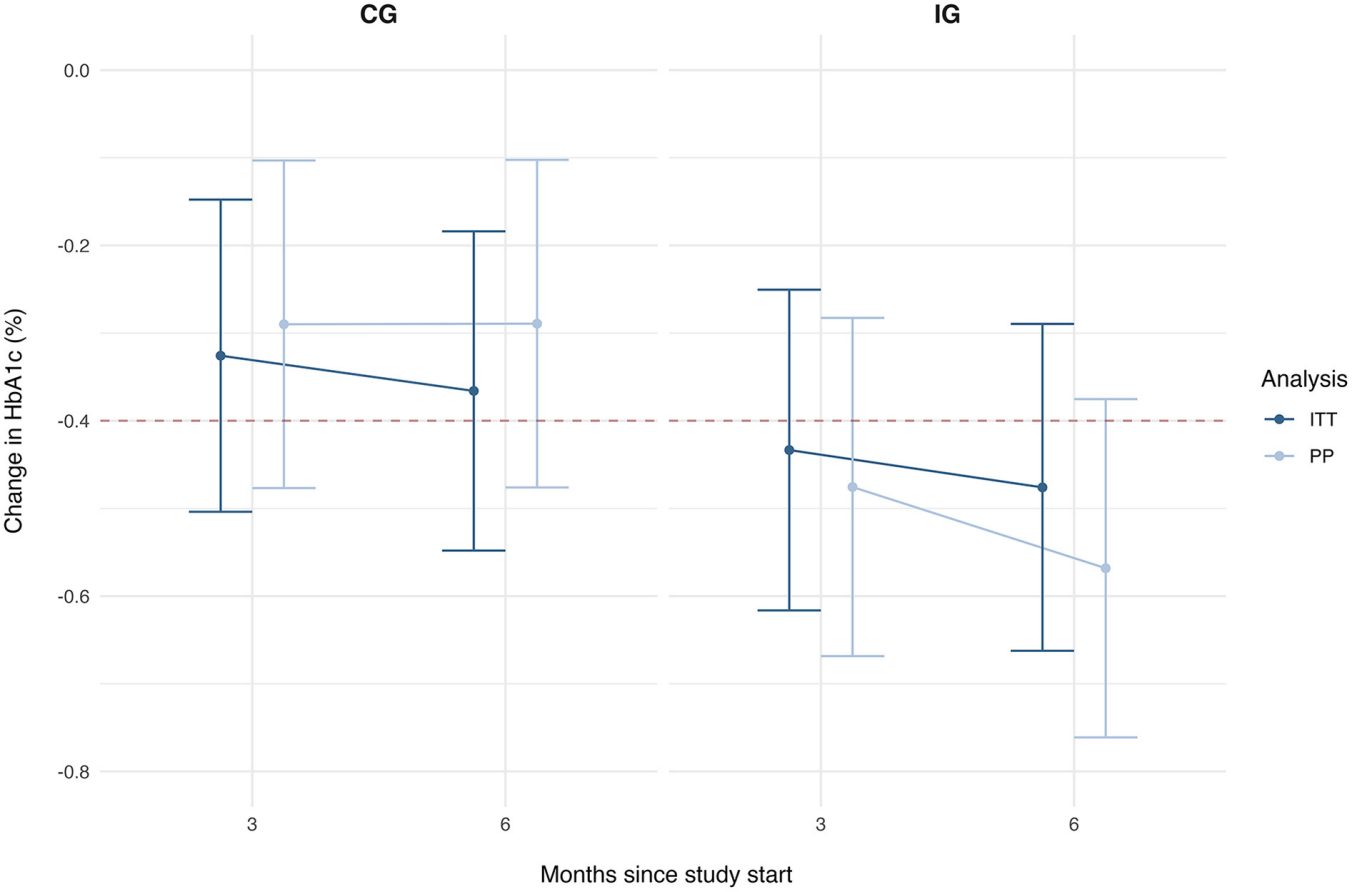
Estimated marginal means and 95% confidence intervals for the HbA1c reduction over time by group and analysis.

The average reduction in HbA1c values based on ITT analysis was −0.48% points after 6 months [95% CI (−0.66; −0.29)], which was also higher compared with the CG [mean difference: −0.12% points, 95% CI (−0.36; 0.14)] (Table 4 and Figure 2). Based on the PP analysis, the reduction the IG achieved an average reduction of difference: −0.57% points [95% CI (−0.76; −0.38)], leading to a higher reduction of almost 0.3% compared with the CG [mean difference: −0.28% points, 95% CI (−0.54; −0.02)].

**Table 4:** Estimated marginal means and 95% confidence intervals for the HbA1c reduction by analysis.

Analysis	Group	Visit 2 (90 d ± 30)	Visit 3 (180 d ± 60)
ITT	IG	−0.43 [−0.62; −0.25]	−0.48 [−0.66; −0.29]
ITT	CG	−0.33 [−0.50; −0.15]	−0.37 [−0.55; −0.18]
PP	IG	−0.48 [−0.67; −0.28]	−0.57 [−0.76; −0.38]
PP	CG	−0.28 [−0.46; −0.10]	−0.29 [−0.48; −0.11]

CG, control group; ITT, intention-to-treat; PP, per-protocol.

The statistical analysis did not yield significant main effects, but only a trend significance for group in the PP analysis (Supplementary Table S5). Both analyses showed significant effects for the baseline HbA1c and indication indicating that the change in HbA1c was more pronounced with higher baseline HbA1c levels and for people with T2D; yet did not significantly differ between the groups. Accordingly, the tests for the hypothesis regarding the changes in HbA1c yielded a significant result for the PP analysis only (see Table 5). In conclusion, based on the PP sample, the IG achieved a clinically relevant and significant reduction in HbA1c levels, which was also significantly higher compared with SoC in Germany.

**Table 5:** Results of the hypothesis testing for the primary and secondary endpoints after 6 months.

Analysis	Hypothesis	Estimate (95% CI)	*t*-statistic	df	*p*_(FDR)_	Cohens *d*
HbA1c (in %)						
ITT	IG vs. CG	−0.12 [−0.36; 0.14]	−0.934	218	0.351	−0.10 [−0.34, 0.13]
ITT	IG	−0.48 [−0.66; −0.29]	−0.773	214	0.220	−0.61 [−0.85, −0.37]
PP	IG vs. CG	−0.28 [−0.54; −0.02]	−2.118	188	0.036*	−0.31 [−0.60, −0.02]
PP	IG	−0.57 [−0.76; −0.38]	−1.721	184	0.044*	−0.86 [−1.16, −0.55]
Diabetes-related distress						
ITT	IG vs. CG	−1.04 [−4.30, 2.21]	−0.631	198	0.529	−0.09 [−0.37, 0.19]
ITT	IG	−1.50 [−4.01; 1.01]	−1.179	198	0.529	−0.17 [−0.45, 0.11]
PP	IG vs. CG	−2.68 [−5.89, 0.54]	−1.647	136	0.102	−0.28 [−0.62, 0.06]
PP	IG	−2.93 [−5.35; −0.51]	−2.396	136	0.009*^a^	−0.41 [−0.75, −0.07]
Well-being						
ITT	IG vs. CG	0.99 [−0.22, 2.19]	1.641	198	0.107	0.23 [−0.05, 0.51]
ITT	IG	0.60 [−0.35; 1.54]	1.249	198	0.107	0.18 [−0.10, 0.46]
PP	IG vs. CG	1.91 [0.59, 3.24]	2.867	137	0.005*^a^	0.49 [0.15, 0.83]
pp	IG	1.15 [0.16; 2.14]	2.293	137	0.012*^a^	0.39 [0.05, 0.73]

CG, control group; df, degrees of freedom; HbA1c, glycated hemoglobin; ITT, intention-to-treat; *p*_(FDR)_, *p*-value (adjusted with the false discovery rate); PP, per-protocol.

Significance codes: 0 ‘***’ 0.001 ‘**’ 0.01 ‘*’ 0.05 ‘.’ 0.1 ‘ ’ 1.

^a^ Due to hierarchical testing to control for multiple testing, the hypothesis tests and respective results based on the ITT analyses for the secondary endpoints are exploratory. Only the test for diabetes-related distress based on the PP analyses can be considered confirmatory.

### Treatment goals and (severe) hypoglycemic events

After 6 months, six (17) participants in the IG and three (8) participants in the CG achieved the treatment goal of an HbA1c of 6.5% (7%). This translates into 5.8% (16.3%) of the initially randomized IG and 3% (8%) of the initially randomized CG. Overall, the probability of achieving the treatment goals of HbA1c values of 6.5% and 7% was higher, yet not statistically significant in the IG (see Supplementary Table S6).

At each visit, the participants were asked about the occurrence of (severe) hypoglycaemic events (summarized in Supplementary Table S6). Overall, the statistical parameters indicate no difference between the patient-reported occurrence of (severe) hypoglycemic events between the groups.

### Changes in well-being and diabetes-related distress

The average change in secondary endpoints demonstrates an improvement in well-being and a decrease in diabetes-related distress in the IG that is more pronounced compared to the CG (Table 6).

**Table 6:** Estimated marginal means and 95% confidence intervals for the secondary endpoints well-being and diabetes-related distress by analysis.

Analysis	Endpoint	Group	Visit 3 (180 d ± 60)
ITT	Well-being	IG	0.54 [−0.21; 1.30]
ITT	Well-being	CG	−0.33 [−1.02; 0.35]
ITT	Diabetes-related distress	IG	−1.50 [−4−01; 1.01]
ITT	Diabetes-related distress	CG	−0.46 [−2.78; 1.86]
PP	Well-being	IG	1.05 [0.07; 2.04]
PP	Well-being	CG	−0.70 [−1.64; 0.24]
PP	Diabetes-related distress	IG	−2.91 [−5.28; −0.54]
PP	Diabetes-related distress	CG	−0.42 [−2.68; 1.84]

CG, control group; ITT, intention-to-treat; PP, per-protocol.

The results of the statistical analyses indicate that the changes are more pronounced (stronger increase in well-being and decrease in diabetes-related distress), the higher the well-being and the lower the diabetes-related distress were at baseline (Supplementary Table 5). Based on the PP analysis, the statistical analysis also indicated that the IG improved their well-being more compared with the CG.

### Additional outcomes

Further beneficial improvements in the IG were observed for the exploratory outcomes, such as weight (in %), waist circumference (in cm), self-management, and quality of life (Supplementary Table S7, S2). This effect was also stronger in the intervention group than in the control group.

## Discussion

The DAVOS trial intended to evaluate if the use of ESYSTA leads to better glycemic control, expressed as a decrease in HbA1c levels (in%) by an enhanced patient self-management. In the ITT and the PP sample, the IG achieved a reduction in HbA1c levels of on average >0.4%. The HbA1c reduction achieved in the IG was more pronounced compared with the CG receiving SoC according to DMP. The results of the PP analyses are better than those of the ITT, allowing us to confirm the hypotheses to test changes in HbA1c levels. First, the IG achieved a clinically relevant reduction of at least 0.4%. Second, this reduction is significantly more pronounced compared with a CG with SoC. Protocol deviations in both groups, such as the occurrence of comorbidities with complex treatment regimens that might have superimposed the treatment effects in both study arms, may serve as a potential explanation for the fact that the hypotheses could not be confirmed in the ITT sample. Protocol deviations also resulted from the corona pandemic that caused organizational obstacles for both study centers and patients.

Studies evaluating programs comparable to ESYSTA, emphasizing blood glucose monitoring for enhancing patient self-management through structured data visualization accessible to both patients and healthcare teams, are scarce. Particularly in the context of digital health applications (DiGA) in Germany, few studies have explored their effects specifically in insulin-treated people with diabetes (23, 24). One RCT that included German, insulin-treated T2D patients showed very similar results: an average HbA1c reduction in the IG of −0.5% (*p* < 0.001, *d* = 0.18) and significant group differences in favor of the IG compared with a CG with DMP care [M*diff*: −0.2%, 95% CI (−0.36%; −0.04%), *p* = 0.013, *d* = 0.16] after 6 months (25). The same *integrated personalised diabetes management* program was analyzed in a one-arm study with American T1D and T2D patients who are treated with insulin and showed the same average reductions after 6 months of 0.5% ± 0.6% (*p* = 0.045, *d* = 0.81) for patients in specialist care settings (26). Many studies, in non-German populations show HbA1c reductions of 0.4% with small to medium effect size (*d* between 0.3 and 0.6) after 3, 4, or 9 months in the IG (27–29) or less-pronounced, non-significant HbA1c reductions in the IG after 3–6 months of 0.2% (30, 31). Evidence further suggests that short-term interventions tend to show higher reductions in HbA1c levels and that the main reductions in long-term studies are achieved in the first three months (25, 29, 32, 33). The results of the DAVOS show, on average, a clinically significant reduction of 0.4% already after the first 3 months. However, compared with other studies, the HbA1c levels continue to decrease over time, leading to HbA1c reductions in patients using ESYSTA after 6 months of almost 0.5%–0.6%. This reduction is further in the range of achieved reductions of some oral diabetes medications (34). While the CG with SoC also shows reductions over time, they are lower, i.e., a clinically relevant reduction of 0.4% is not achieved on average during the trial duration.

An HbA1c below 7% is a general target goal recommended in guidelines for diabetes to prevent macrovascular complications. In the DAVOS trial, twice as many IG participants (16% and 6%), compared with CG participants (8% and 3%), reached the target of 7% and 6.5%, respectively. According to guidelines, a tighter HbA1c goal with values below 6.5% is recommended for younger participants with recent diagnosis, while less-stringent goals (HbA1c <8% or even <9%) might be more suitable for older participants with longer disease durations. As the study population on average falls more in the latter category, the adaptability of treatment goals <7% is only partly given (3, 8). Generally, HbA1c treatment goals are highly individualized and should be in accordance with sociodemographic characteristics and comorbidities. In fact, landmark studies such as the Diabetes Control and Complications Trial (DDCT) or the UK Prospective Diabetes Study (UKPDS) show positive correlations between reductions in HbA1c levels and the long-term risk reduction of microvascular complications without specified thresholds (35–37). Individualized HbA1c treatment goals rather than predefined thresholds such as 7% are also used in the evaluation of DMPs in Germany (38).

### Secondary and exploratory outcomes

Apart from improvements in the primary outcome, HbA1c levels, the DAVOS trial further shows improvements in diabetes-associated behavioral and health outcomes. The secondary outcomes, well-being, and self-management in the IG improved, while diabetes-associated distress decreased. In particular, the PP analyses show a significant positive effect of ESYSTA on well-being and diabetes-related distress, even in comparison with the CG with SoC. Positive trend for both these secondary endpoints in the IG is also present in the ITT analyses, while in the CG with DMP, well-being tends to deteriorate, and diabetes-related distress remains unchanged. Improvements in the IG are also present in the exploratory outcomes, quality of life, and self-management, while the self-management in the CG with DMP care remains unchanged. The increase in quality of life was also present for the CG, but the change ranges within minimal clinically important differences for the IG only and can thus be considered relevant (39). Since many studies do not report secondary outcomes, the evidence for well-being, quality of life, and diabetes-related distress is generally limited. Studies investigating the change in quality of life either report non-significant but numerical improvements or no changes (26, 29, 31, 32). In the present DAVOS trial, most participants reported low levels of diabetes-related distress and no impaired well-being at study start, too, making improvements difficult to achieve (40). Yet, the IG improved their well-being and reduced their diabetes-related distress further, especially T2D patients. The potential to optimize the management of a chronic disease, i.e., lowering the disease burden (e.g., reduced HbA1c levels and improved blood glucose control), is correlated with improvements in quality of life, well-being, or the reduction in diabetes-related distress (14, 41). However, it is assumed that such secondary treatment outcomes are more indirectly affected by the intervention itself but rather moderated by improvements in the primary outcome and the reduced disease progression and associated complications associated with it (42, 43). As a result, the improvement in secondary and exploratory outcomes in the DAVOS trial indicates not only an improvement in well-being/quality of life, diabetes-related distress, and self-management but also the disease-related burden.

Current diabetes therapy guidelines emphasize the importance of weight reduction, particularly for T2D. For people with diabetes who are treated with insulin, the interventional focus shifts from a simple tablet intake (oral antidiabetics) to insulin application requiring stronger self-management skills. This is crucial to prevent both hyperglycemia and hypoglycemia and to optimize long-term outcomes. Also, due to the nature of T1D, some patients may need to focus on weight gain rather than weight loss to reach a healthy body weight. Such developments and shifting therapy goals are also reflected in other studies. While programs that focus on lifestyle interventions in T2D patients sometimes show decreases in weight (23, 44), studies with people with diabetes who are treated with insulin usually indicate weight stability over time (29, 32). Patients in the DAVOS showed non-significant changes in weight in both groups and in waist circumference, respectively.

Treatment guidelines already increasingly emphasize the importance of digital diabetes devices such as CGMs, insulin pumps, pens, and digital monitoring systems (10). Yet, the main barriers to the usage of smart devices are the lack of interoperability, either with glucose analysis software or third-party apps (45, 46). ESYSTA offers a diabetes management tool incorporating a variety of devices. It collects, stores, and visualizes data in an easy-to-understand way. Especially, the daily and blood glucose profiles and the integration of the traffic light display in the ESYSTA dashboard to illustrate risks (see Supplementary Figures S1–S3) have been shown to be highly accepted by patients and health care professionals in other studies (47). The results of the user experience questionnaire in the DAVOS trial suggest its comprehensibility is a particular strength of ESYSTA (52, 53). This is also reflected in the drop-out behaviour in the IG, which does not seem to be connected to dissatisfaction with the digital health application. Non-use of ESYSTA in the IG seemed to have been connected to a lack of support from health care professionals in initiating the treatment.

### Strengths and limitations

One of the main limitations in studies analyzing digital health applications, as well as the DAVOS trial, is the lack of a placebo and blinding in the study design that might introduce bias, especially when it comes to patient-reported outcomes (48). However, the design of a potential placebo or sham app has its own challenges and will only control for certain features of the analyzed health application (49). Moreover, in case of diabetes, a recent review suggests that while secondary outcomes, such as well-being or disease burden, might be affected by patients’ expectation, placebos do not seem to have an effect on the pathophysiology, i.e., HbA1c values (50). The setting of the DAVOS trials was designed and set very close to real-world conditions, e.g., participants were part of the DMP programs for diabetes to ensure consistent and equal treatment (SoC) in both the IG and the CG. This also leads to primary endpoint measurements taken and documented within the standard of care, leading to an increase in external and internal validity and lowering potential measurement biases. Also, the drop-out, which is lower than expected and lower or comparable to other studies (26, 32), shows in fact that the study settings do not lead to patients dropping out since no complex and time-consuming additional study procedures are introduced. Although the study was close to real-life conditions, the context of the coronavirus pandemic and the associated organizational barriers may have limited external validity. In the context of the study, it can be assumed that participants were particularly motivated. Combined with the knowledge about the group assignment, patients in the control group might have been particularly led to use of alternative treatment options, including effective diabetes medications such as GLP-1 receptor agonists. To address this potential bias, evidence from real-world studies, e.g., studies based on data from registries or user data, should complement findings from controlled trials. Even after the exclusion of additional protocol deviations, the number of excluded patients is below the expected 30%. Importantly, the study population is representative of the expected target population, i.e., middle-aged and older patients in a broad range of HbA1c values at baseline (7.5%–11%), well-balanced between men and women. Regarding the age and gender distribution, the study populations are transferable to the German healthcare context (38, 51). Nevertheless, the results indicate that patients might profit differently depending on their indication. Also, the present study does not take into account the actual usage behavior of the IG. Both aspects should be looked into in secondary analyses.

## Conclusion

The evidence presented with DAVOS shows that ESYSTA leads to improved blood glucose control in people with diabetes treated with insulin. The positive impact of ESYSTA on HbA1c values is further supported by patients’ reported outcomes, well-being, quality of life, self-management, and diabetes-related distress that improved in the IG. As such, ESYSTA is in line with current quality criteria of diabetes DMPs, which are evaluated based on structural improvements including the prevention and avoidance of secondary diseases (38, 51). Being an easy-to-understand digital self-management tool that also offers the inclusion of data generated by other digital diabetes devices, ESYSTA has the potential to improve digital diabetes management.

## Data Availability

The original contributions presented in the study are included in the article/Supplementary Material, further inquiries can be directed to the corresponding author.
