# Supplementary material for: Impact of a digital application on HbA1c levels in people with diabetes: a randomized controlled trial
Source: Front Digit Health. 2025 Jun 30;7:1544668. doi: 10.3389/fdgth.2025.1544668 (PMC12257309; doi:10.3389/fdgth.2025.1544668)

Supplementary Tables and Figures

Supplementary Table 1: Description of Functions of ESYSTA® APP and portal. 2

Supplementary Table 2: Number and percentage of patients reporting levels 1 to 5 by dimension of the EQ-5D-5L by group and visit, based on the ITT sample. 4

Supplementary Table 3: Baseline values of the subscales of the self-management questionnaire (SDSCA-G) based on the ITT sample. 5

Supplementary Table 4: Demographic and endpoint characteristics of the per-protocol population at baseline. 6

Supplementary Table 5: Results of the ANCOVA for the primary and secondary endpoints by analysis after 6 months of treatment. 7

Supplementary Table 6: Estimated Odds Ratios, Risk Ratios and Risk Differences and 95% confidence intervals for the proportion of participants achieving HbA1c treatment goals (6.5% / 7%) and respective p-values for group differences after 6 months of treatment. 8

Supplementary Figure 1: ESYSTA® Web-Portal 7-day Blood glucose Trend (left) and Traffic Light Display to highlight Hypo- and Hyperglycemic events (right) for several a fictitious patient. 10

Supplementary Figure 2: ESYSTA® Web-Portal 7-day Blood glucose Trend for several fictitious patients (Health Care Professional View). 11

Supplementary Figure 3: ESYSTA® App 7-day Blood glucose Trend (left) and diary including blood glucose, insulin (in IE) and carbs (right). 12

Supplementary Figure 4: Distribution per Item of the User Experience Questionnaire after 6 months of treatment based on responses by 73 intervention group patients (70.2%). 13

Supplementary Figure 5: Mean and Variance of the User Experience Categories after 6 months of treatment based on responses by 73 intervention group patients (70.2%). 14

Supplementary Table 1: Description of Functions of ESYSTA® APP and portal.

ESYSTA® APP	ESYSTA® PORTAL
- automatic data synchronization with the ESYSTA® PORTAL<br>- import of blood glucose and insulin data simply via Bluetooth®.<br>- manual recording of blood glucose readings, insulin doses and bread units<br>- fully automatic synchronization of limit values, insulin type used and measurement units (mmol/l and mg/dl)<br>- display of diary data when no suitable computer is available to access the ESYSTA® PORTAL, e. g. on vacation or during doctor consultations<br>- detailed daily display of blood glucose, insulin and bread units in a clearly arranged timeline diary<br>- blood glucose history in three- and seven-day view<br>- integrated diagram with insulin doses and blood glucose values<br>- clear data evaluation/target range analysis<br>- simplified display and preparation of selected therapy data for optimization and support of patient self-management<br>- quick overview of the metabolic setting by the ESYSTA® Assistant with the ESYSTA® traffic light<br>- marking of blood glucose readings for assignment to meals (fasting, pre- and postprandial and other selections)<br>- also, usable when offline	- worldwide usable, easy web access to therapy data via web browser<br>- data preparation in clear tables and graphics – complete and without gaps<br>- support for automated import of data from suitable blood glucose meters and insulin pens<br>- detailed daily display of blood glucose, insulin and bread units in a clearly arranged diary<br>- daily blood glucose history in seven-day view<br>- integrated diagram display of insulin doses and blood glucose values<br>- clear data evaluation/target range analysis<br>- marking of blood glucose readings to set them into context with meals (fasting, pre- and postprandial and further selection options)<br>- daytime analysis of blood glucose values<br>- simplified display of selected therapy data for optimization and support of patient self-management<br>- data access for the physician possible<br>- quick overview of the metabolic setting by the ESYSTA® APP with specially developed traffic light function supporting the patient in his/her therapy (empowerment) and gives the physician indications of a possible need for intervention<br>- protected message exchange with the physician<br>- display of relevant parameters for each patient and medical professional

	- entry of further medical or laboratory data in the form of an electronic patient file is possible<br>-

Supplementary Table 2: Number and percentage of patients reporting levels 1 to 5 by dimension of the EQ-5D-5L by group and visit, based on the ITT sample.

	**Baseline**	**Visit 3 (180d ± 60)**	**Baseline**	**Visit 3 (180d ± 60)**
	**Intervention Group**		**Control Group**	
	(n = 104)	(n = 84)	(n = 100)	(n = 88)
Mobility				
1	70 (67.3%)	59 (70.2%)	58 (58.0%)	58 (65.9%)
2	12 (11.5%)	11 (13.1%)	20 (20.0%)	12 (13.6%)
3	18 (17.3%)	10 (11.9%)	15 (15.0%)	13 (14.8%)
4	3 (2.9%)	4 (4.8%)	7 (7.0%)	5 (5.7%)
5	1 (1.0%)	0 (0%)	0 (0%)	0 (0%)
Self-Care				
1	99 (95.2%)	80 (95.2%)	93 (93.0%)	84 (95.5%)
2	1 (1.0%)	2 (2.4%)	6 (6.0%)	2 (2.3%)
3	2 (1.9%)	1 (1.2%)	1 (1.0%)	2 (2.3%)
4	1 (1.0%)	1 (1.2%)	0 (0%)	0 (0%)
5	1 (1.0%)	0 (0%)	0 (0%)	0 (0%)
Usual Activities				
1	85 (81.7%)	72 (85.7%)	76 (76.0%)	70 (79.5%)
2	12 (11.5%)	5 (6.0%)	20 (20.0%)	8 (9.1%)
3	4 (3.8%)	5 (6.0%)	3 (3.0%)	7 (8.0%)
4	2 (1.9%)	2 (2.4%)	1 (1.0%)	3 (3.4%)
5	1 (1.0%)	0 (0%)	0 (0%)	0 (0%)
Pain / Discomfort				
1	33 (31.7%)	31 (36.9%)	29 (29.0%)	29 (33.0%)
2	34 (32.7%)	29 (34.5%)	34 (34.0%)	33 (37.5%)
3	23 (22.1%)	18 (21.4%)	24 (24.0%)	15 (17.0%)
4	13 (12.5%)	5 (6.0%)	13 (13.0%)	10 (11.4%)
5	1 (1.0%)	1 (1.2%)	0 (0%)	1 (1.1%)
Anxiety / Depression				
1	61 (58.7%)	53 (63.1%)	66 (66.0%)	57 (64.8%)
2	23 (22.1%)	15 (17.9%)	15 (15.0%)	14 (15.9%)
3	9 (8.7%)	12 (14.3%)	10 (10.0%)	13 (14.8%)
4	9 (8.7%)	4 (4.8%)	6 (6.0%)	3 (3.4%)
5	2 (1.9%)	0 (0%)	3 (3.0%)	1 (1.1%)

Supplementary Table 3: Baseline values of the subscales of the self-management questionnaire (SDSCA-G) based on the ITT sample.

	IG (n=104)	CG (n=100)	Overall (N = 204)
**Overall Scale**			
Mean (SD)	4.02 (0.995)	4.15 (1.05)	4.08 (1.02)
Median [Min, Max]	4.05 [1.60, 6.20]	4.10 [1.30, 6.80]	4.10 [1.30, 6.80]
**Diet**			
Mean (SD)	3.97 (1.53)	4.15 (1.50)	4.06 (1.51)
Median [Min, Max]	4.13 [0.250, 7.00]	4.25 [1.00, 7.00]	4.25 [0.250, 7.00]
**Activity**			
Mean (SD)	3.25 (1.68)	3.07 (1.78)	3.16 (1.72)
Median [Min, Max]	3.50 [0, 7.00]	3.50 [0, 7.00]	3.50 [0, 7.00]
**Blood Sugar**			
Mean (SD)	6.19 (1.87)	6.48 (1.33)	6.33 (1.63)
Median [Min, Max]	7.00 [0, 7.00]	7.00 [0, 7.00]	7.00 [0, 7.00]
**Footcare**			
Mean (SD)	2.71 (2.33)	2.88 (2.39)	2.79 (2.36)
Median [Min, Max]	3.00 [0, 7.00]	3.50 [0, 7.00]	3.50 [0, 7.00]

Note: CG, control group; IG, intervention group; Min, minimum; Max, maximum; SD, standard deviation.

Supplementary Table 4: Demographic and endpoint characteristics of the per-protocol population at baseline.

	**IG** (n=71)	**KG** (n=72)	**Overall** (N=143)
**Age**			
Mean (SD)	56.0 (13.4)	58.0 (13.3)	57.0 (13.3)
Median [Min, Max]	59.0 [18.0, 84.0]	60.5 [26.0, 91.0]	60.0 [18.0, 91.0]
**Gender**			
Male	43 (60.6%)	41 (56.9%)	84 (58.7%)
Female	28 (39.4%)	31 (43.1%)	59 (41.3%)
**Type of Diabetes**			
T1DM	21 (29.6%)	26 (36.1%)	47 (32.9%)
T2DM	50 (70.4%)	46 (63.9%)	96 (67.1%)
**Years Since Diagnosis**			
Mean (SD)	14.9 (10.1)	16.2 (9.38)	15.6 (9.74)
Median [Min, Max]	13.0 [0, 43.0]	15.5 [0, 45.0]	14.0 [0, 45.0]
**HbA1c (in %)**			
Mean (SD)	8.44 (0.776)	8.44 (0.809)	8.44 (0.790)
Median [Min, Max]	8.20 [7.50, 10.8]	8.25 [7.50, 10.8]	8.20 [7.50, 10.8]
**WHO**			
Mean (SD)	15.1 (5.52)	16.0 (5.57)	15.6 (5.54)
Median [Min, Max]	16.0 [2.00, 24.0]	16.5 [2.00, 25.0]	16.0 [2.00, 25.0]
**PAID**			
Mean (SD)	15.1 (16.5)	12.8 (12.8)	14.0 (14.7)
Median [Min, Max]	8.75 [0, 75.0]	7.50 [0, 52.5]	7.50 [0, 75.0]
**Weight (in kg)**			
Mean (SD)	92.2 (19.3)	92.5 (19.8)	92.4 (19.5)
Median [Min, Max]	92.0 [56.0, 150]	91.1 [57.5, 161]	92.0 [56.0, 161]
**BMI (in kg/m^2)**			
Mean (SD)	30.0 (5.24)	31.0 (5.34)	30.5 (5.30)
Median [Min, Max]	30.8 [19.6, 39.9]	30.9 [18.3, 39.9]	30.8 [18.3, 39.9]
**Waist-Cicumference**			
Mean (SD)	109 (14.4)	109 (17.8)	109 (16.1)
Median [Min, Max]	109 [74.0, 145]	106 [74.0, 151]	108 [74.0, 151]
Missing	3 (4.2%)	5 (6.9%)	8 (5.6%)

Note: BMI, body mass index; CG, control group; HbA1c, glycated hemoglobin; IG, intervention group; Min, minimum; Max, maximum; SD, standard deviation; T1DM/T2DM, diabetes mellitus type 1/2.

Supplementary Table 5: Results of the ANCOVA for the primary and secondary endpoints by analysis after 6 months of treatment.

Analysis	Factor	df	X^2-*statistic*	*p-value*
		HbA1c (in %)		
ITT	group	1	0.733	0.392
ITT	time	1	0.3089	0.579
ITT	group*time	1	0.000	0.982
ITT	Baseline HbA1c	1	61.874	< .0001***
ITT	Indication	1	6.332	0.012*
ITT	Gender	1	0.795	0.373
PP	group	1	3.6969	0.055.
PP	time	1	0.7574	0.384
PP	group*time	1	1.990	0.374
PP	Baseline HbA1c	1	0.7916	< .0001***
PP	Indication	1	5.7199	0.017*
PP	Gender	1	0.293	0.588
		well-being		
ITT	group	1	2.606	0.108
ITT	baseline well-being	1	37.141	< .0001***
ITT	baseline HbA1c	1	2.040	0.155
ITT	indication	1	1.696	0.195
ITT	gender	1	0.662	0.417
PP	group	1	8.218	0.005**
PP	baseline well-being	1	34.603	< .0001***
PP	baseline HbA1c	1	1.634	0.203
PP	indication	1	1.002	0.319
PP	gender	1	0.068	0.795
		diabetes-related distress		
ITT	group	1	0.398	0.529
ITT	baseline distress	1	32.841	< .0001***
ITT	baseline HbA1c	1	0.155	0.694
ITT	indication	1	0.130	0.719
ITT	gender	1	2.028	0.156

**PP**	**group**	1	2.714	0.102
**PP**	**baseline distress**	1	27.215	< .0001***
**PP**	**baseline HbA1c**	1	0.881	0.350
**PP**	**indication**	1	0.002	0.963
**PP**	**gender**	1	0.271	0.603

Note: CG, control group; df, degrees of freedom; HbA1c, glycated hemoglobin; IG, intervention group.

*Significance Codes: 0 '***' 0.001 '**' 0.01 '*' 0.05 '.' 0.1 ' ' 1*

Supplementary Table 6: Estimated Odds Ratios, Risk Ratios and Risk Differences and 95% confidence intervals for the proportion of participants achieving HbA1c treatment goals (6.5% / 7%) and respective p-values for group differences after 6 months of treatment.

	Estimate and 95% confidence intervals	p-value
**6.5%**		
Odds Ratio	1.97 [0.41; 12.54]	0.499
Risk Ratio	1.33 [0.82; 2.15]	0.499
Risk Difference	0.03 [-0.03; 0.08]	0.499
**7%**		
Odds Ratio	2.24 [0.86; 6.31]	0.088.
Risk Ratio	1.40 [1.03; 1.90]	0.088.
Risk Difference	0.08 [-0.01; 0.17]	0.088.
**Hypoglycaemic Events**		
Odds Ratio	1.12 [0.72; 1.74]	0.588
Risk Ratio	1.06 [0.86; 1.30]	0.588
Risk Difference	-0.02 [-0.08; 0.04]	0.588

*Significance Codes: 0 '***' 0.001 '**' 0.01 '*' 0.05 '.' 0.1 ' ' 1*

Supplementary Table 7: Results of the hypotheses testing for the secondary endpoints after 6 months based on the intention-to-treat sample.

Comp-arison	Estimate (95% CI)	*t-statistic*	*df*	*p-value*	*Cohen's d*
			**Weight (in %)**		
**IG vs. CG**	-0.63 [-4.08; 2.83]	-0.357	393	0.722	-0.04 [-0.23, 0.16]
**IG**	3.38 [0.78; 5.98]	2.554	389	0.916	0.26 [0.06, 0.46]
**CG**	4.01 [1.50; 6.52]	3.139	392	0.999	0.32 [0.12, 0.52]
			**Waist Circumference (in cm)**		
**IG vs. CG**	0.00 [-2.20; 2.21]	0.008	306	0.994	0.00 [-0.22, 0.23]
**IG**	0.24 [-1.39; 1.86]	0.285	301	0.612	0.03 [-0.19, 0.26]
**CG**	0.23 [-1.35; 1.81]	0.283	304	0.611	0.03 [-0.19, 0.26]
			**Self-Management (SDSCA-G)**		
**IG vs. CG**	0.14 [-0.11; 0.38]	1.902	198	0.276	0.16 [-0.12, 0.43]
**IG**	0.12 [-0.07; 0.31]	1.230	198	0.110	0.17 [-0.10, 0.45]
**CG**	-0.02 [-0.19; 0.16]	-0.183	198	0.573	-0.03 [-0.30, 0.25]
			**Quality of life (EQ-5D-5L index)**		
**IG vs. CG**	0.01 [-0.02; 0.05]	0.806	198	0.421	0.11 [-0.16, 0.39]
**IG**	0.04 [0.01; 0.07]	2.880	198	0.002	0.41 [0.13, 0.69]
**CG**	0.03 [0.00; 0.05]	2.037	198	0.022	0.29 [0,01, 0.57]

Note: CG, control group; CI, confidence interval; df, degrees of freedom; HbA1c, glycated hemoglobin; IG, intervention group, quality of life index (range 0-1).

*Significance Codes: 0 '***' 0.001 '**' 0.01 '*' 0.05 '.' 0.1 ' ' 1*

Supplementary Figure 1: ESYSTA® Web-Portal 7-day Blood glucose Trend (left) and Traffic Light Display to highlight Hypo- and Hyperglycemic events (right) for several a fictitious patient.

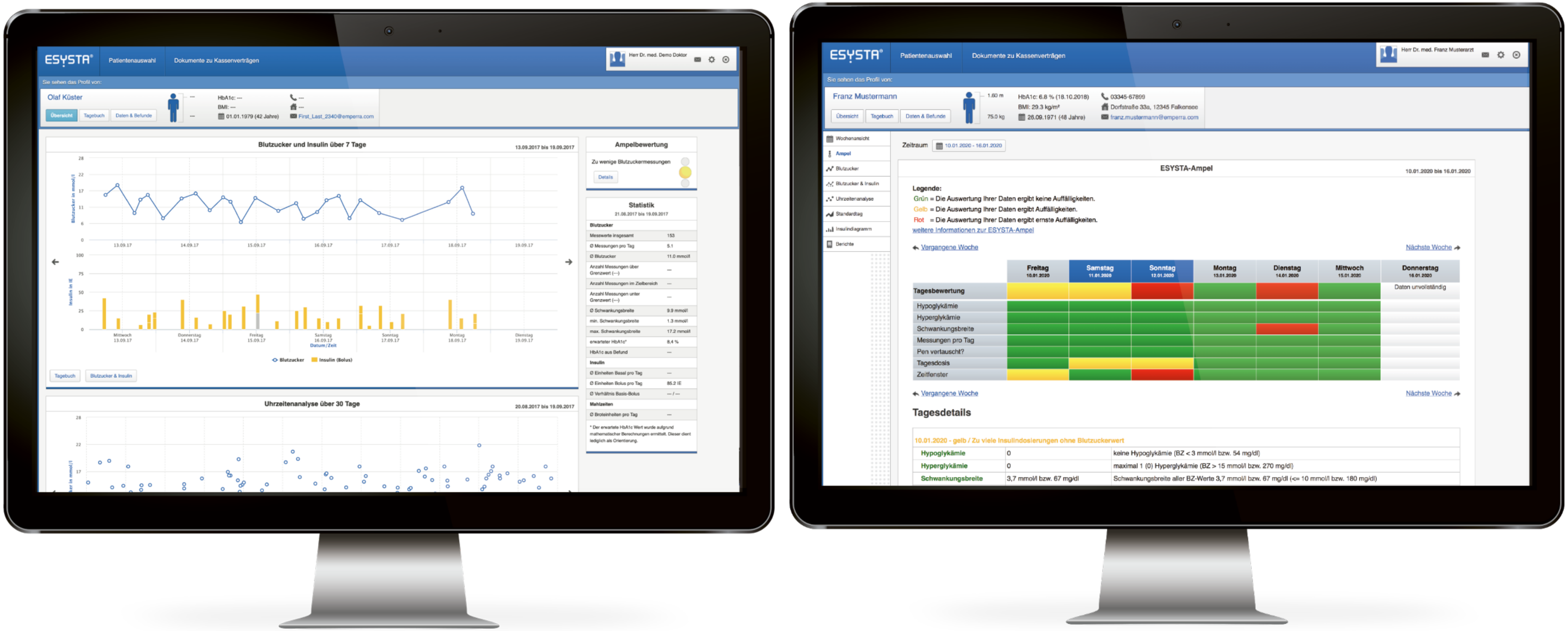

Supplementary Figure 2: ESYSTA® Web-Portal
7-day Blood glucose Trend for several fictitious patients (Health Care Professional View).

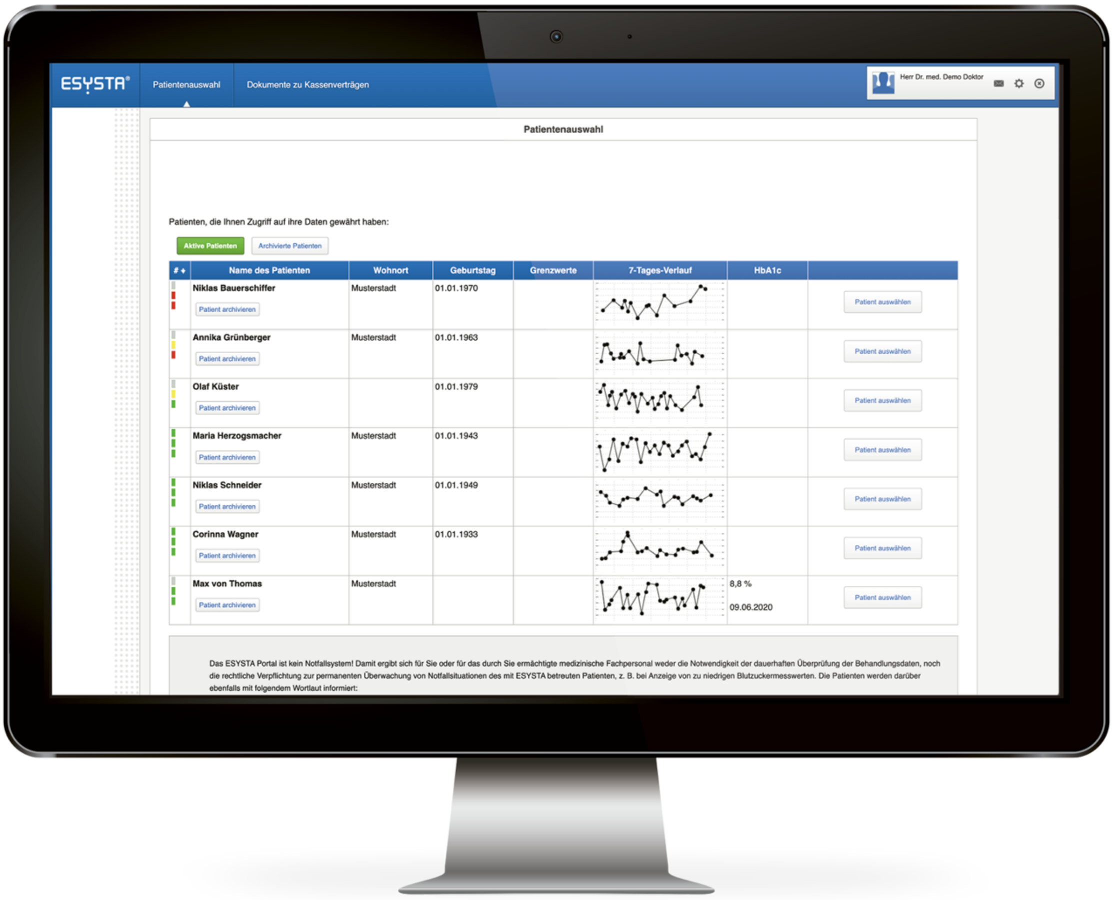

Supplementary Figure 3: ESYSTA® App
7-day Blood glucose Trend (left) and diary including blood glucose, insulin (in IE) and carbs (right).

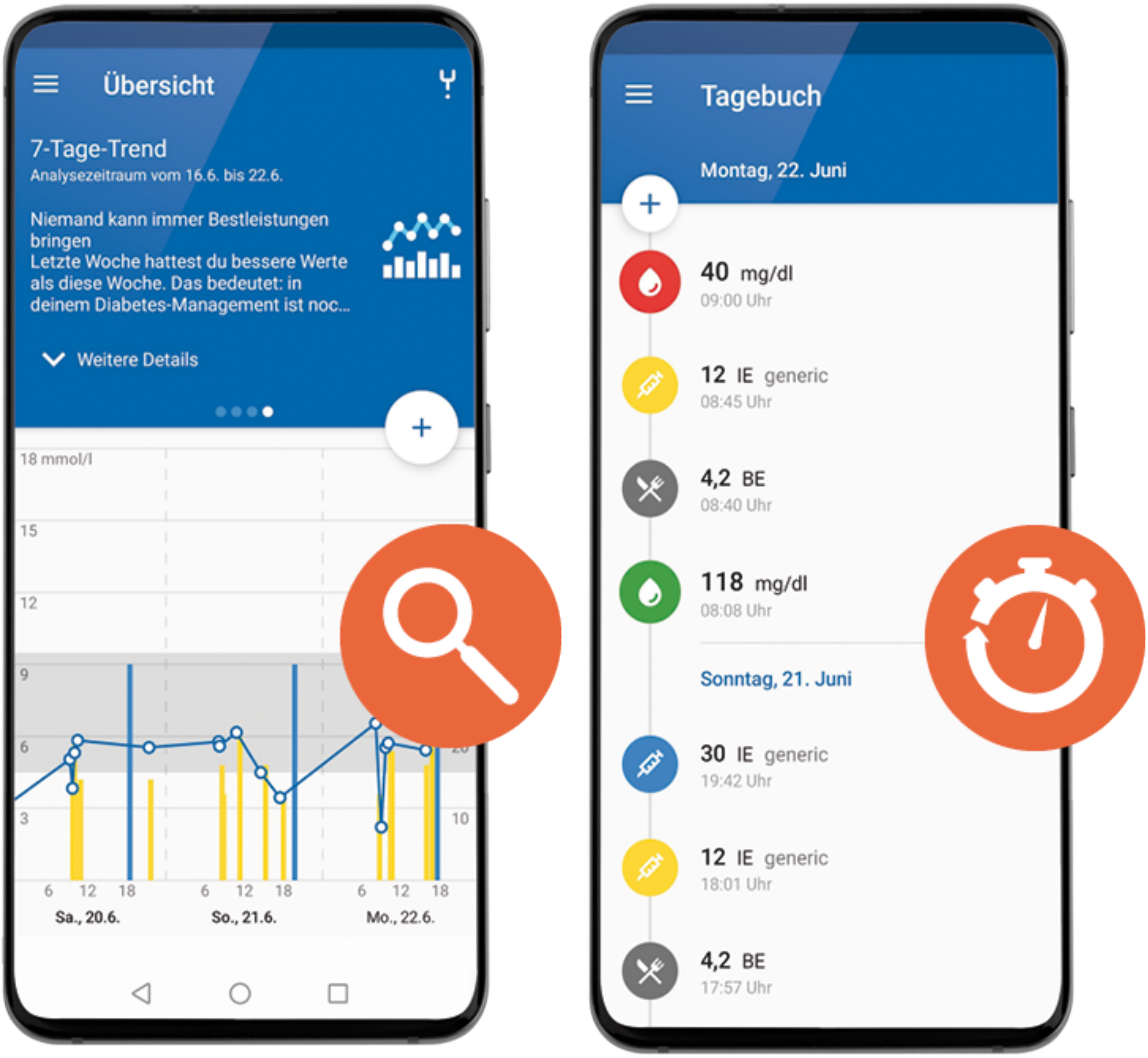

Supplementary Figure 4: Distribution per Item of the User Experience Questionnaire after 6 months of treatment based on responses by 73 intervention group patients (70.2%).

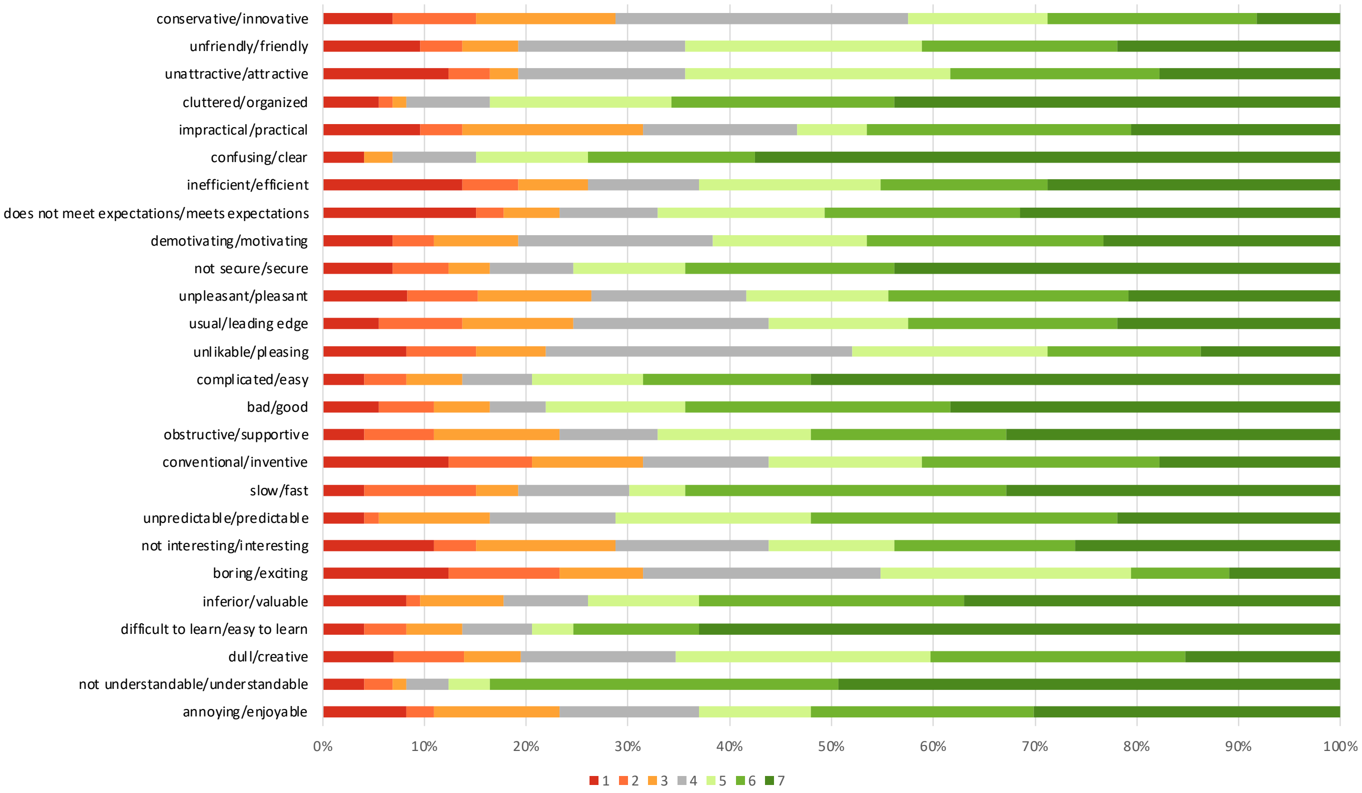

Supplementary Figure 5: Mean and Variance of the User Experience Categories after 6 months of treatment based on responses by 73 intervention group patients (70.2%).

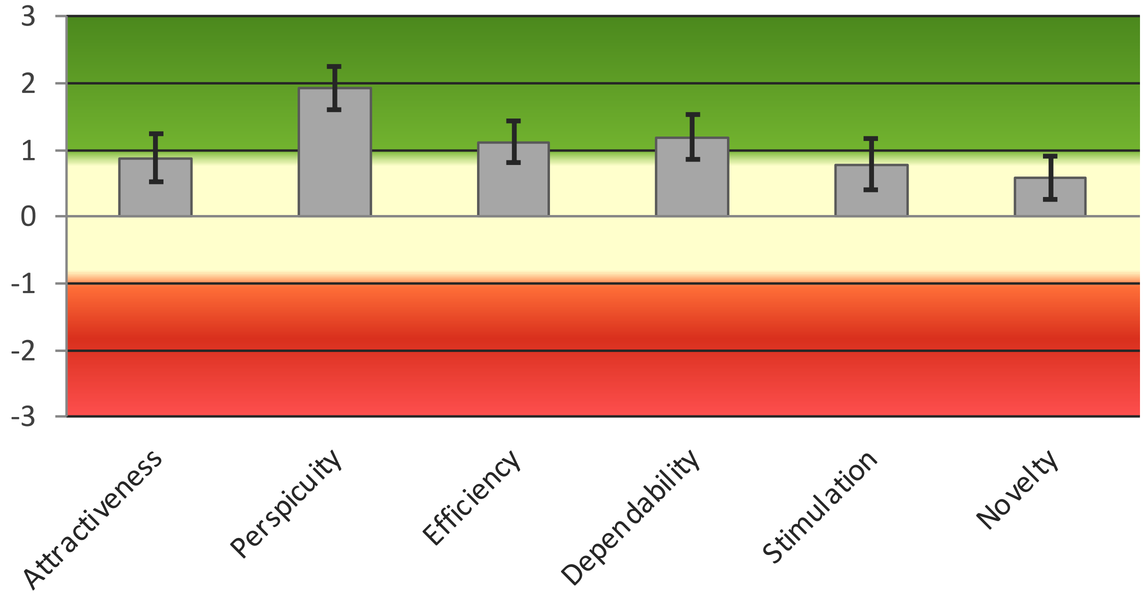

Supplement: Supplementary file 1 [file Table1.pdf]
